# The Emerging Role of Schwann Cells in the Tumor Immune Microenvironment and Its Potential Clinical Application

**DOI:** 10.3390/ijms252413722

**Published:** 2024-12-23

**Authors:** Shan Zhang, Jing Chen, Fanjun Cheng, Fang Zheng

**Affiliations:** 1 Institute of Hematology, Union Hospital, Tongji Medical College, Huazhong University of Science and Technology, Wuhan 430022, China; 2 Department of Pediatrics, Union Hospital, Tongji Medical College, Huazhong University of Science and Technology, Wuhan 430022, China

**Keywords:** Schwann cells, immunocytes, tumor microenvironment

## Abstract

As the primary glial cells in the peripheral nervous system (PNS), Schwann cells (SCs) have been proven to influence the behavior of cancer cells profoundly and are involved in cancer progression through extensive interactions with cancer cells and other stromal cells. Indeed, the tumor microenvironment (TME) is a critical factor that can significantly limit the efficacy of immunotherapeutic approaches. The TME promotes tumor progression in part by reshaping an immunosuppressive state. The immunosuppressive TME is the result of the crosstalk between the tumor cells and the different immune cell subsets, including macrophages, natural killer (NK) cells, dendritic cells (DCs), lymphocytes, myeloid-derived suppressor cells (MDSCs), etc. They are closely related to the anti-tumor immune status and the clinical prognosis of cancer patients. Increasing research demonstrates that SCs influence these immune cells and reshape the formation of the immunosuppressive TME via the secretion of various cytokines, chemokines, and other effector molecules, eventually facilitating immune evasion and tumor progression. In this review, we summarize the SC reprogramming in TME, the emerging role of SCs in tumor immune microenvironment, and the underlying mechanisms involved. We also discuss the possible therapeutic strategies to selectively target SCs, providing insights and perspectives for future research and clinical studies involving SC-targeted treatment.

## 1. Introduction

The tumor microenvironment (TME) is a highly heterogeneous ecosystem involving cancer cells, multiple immunocytes, and many other types of cells, as well as their myriad interactions within the tumor [1,2,3]. The composition and state of the TME vary by tumor type, stage, location, intrinsic characteristics of the cancer cells, and patient-specific factors. They can be either anti-tumor or pro-tumor [1,4,5]. Although the immune system can eliminate tumors through the cancer immune cycle, tumors appear to ultimately evade immune surveillance by shaping the immunosuppressive microenvironment [6,7]. Indeed, glial cells, especially Schwann cells (SCs), also play an important role in cancer development and progression [8,9].

SCs, originating from the neural crest, perform multiple crucial functions in the peripheral nervous system (PNS), such as protecting, supporting, and nourishing nerve fibers; facilitating nerve impulse conduction; initiating pain sensation; and aiding neural development, regeneration, and repair [7,10,11,12,13,14,15]. Upon nerve injury, they undergo extensive morphological and expression changes in a process called “adaptive cellular reprogramming” [16,17,18,19]. In this repair state, SCs re-enter the cell cycle and coordinate the multifaceted process of nerve regeneration, including recruiting immune cells [20,21,22], breaking down myelin debris, remodeling the extracellular matrix (ECM), and expressing factors essential for axon survival, regrowth, and guidance [23,24,25,26]. Recent studies have also demonstrated that SCs participate in forming and regulating epidermal tactile receptors [11]. Additionally, SCs secrete immune-related molecules that modulate innate and adaptive immune responses, contributing to various pathological processes, including cancer [27].

**Figure 1:**
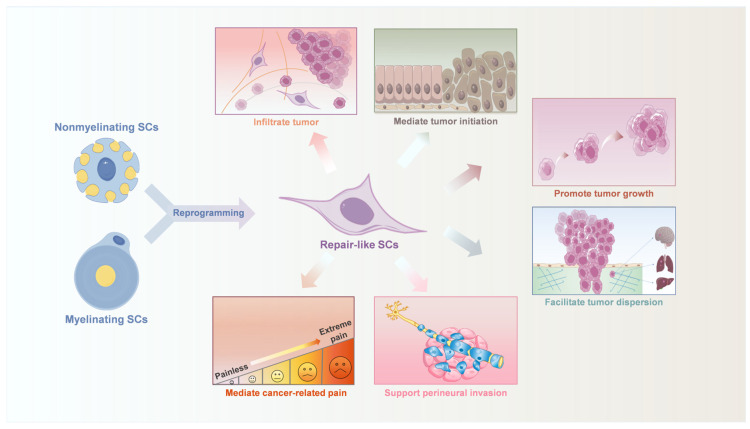
The role of Schwann cells (SCs) in cancer biology. Upon activation by other cells within the tumor microenvironment (TME), SCs undergo reprogramming to a phenotype reminiscent of the repair SCs following nerve injury. These repair-like SCs contribute to cancer progression through multiple mechanisms.

Remarkably, the crosstalk between SCs and tumor cells has attracted much attention in recent decades. SCs exhibit a particular and specific affinity to cancer cells, migrating toward them even before cancer invades nerves [28]. They promote tumor proliferation, guide cancer cells to nerves, and facilitate their dispersion [8,9,29,30]. SCs also form the Tumor-Activated Schwann cell track (TAST) to support cancer cell migration and enhance their motility [31] while promoting epithelial–mesenchymal transition (EMT) of tumor cells [32,33,34,35]. These actions collectively contribute to cancer cell migration, invasion, and perineural invasion (PNI) (Figure 1). Additionally, SCs secrete various bioactive molecules that modulate the phenotype and functions of immunocytes and fibroblasts, establishing an immunosuppressive TME and promoting tumor progression [29,36,37].

In this review, we focus on SCs’ influence on immunocytes within the TME, aiming to identify effective targets that could be leveraged to enhance cancer therapeutic efficacy. Furthermore, we highlight underexplored aspects of SC biology in the TME, which may benefit further research.

## 2. SC Reprogramming in TME

In the TME, SCs are often described as undergoing a reprogramming process analogous to neural repair. Shurin et al. (2019) reported significant alterations in the expression profile of SCs when exposed to melanoma-conditioned medium. Notably, there was a downregulation of genes associated with terminal differentiation and myelination, along with an upregulation of repair SC markers. Functionally, this reprogramming enabled SCs to boost the secretion of factors essential for neural and tissue repair, ECM reorganization, immunomodulation, chemotaxis, and myelin phagocytosis. These changes also enhanced SCs’ ability to catabolize collagen matrix and recruit and induce macrophage polarization [38].

Despite these observations, the precise mechanism underlying this reprogramming of SCs within the TME remains largely elusive. Several studies have suggested that this process is mediated by factors secreted by tumor cells. Deborde et al. demonstrated that SC reprogramming in pancreatic cancer is dependent on c-Jun [31]. In colon cancer, researchers revealed that exosomal miR-21-5p, derived from cancer cells, enhances SC proliferation and migration by stimulating SCs to secrete nerve growth factor (NGF) [39]. Additionally, tumor-associated immune cells can also promote SCs’ reprogramming. In pancreatic ductal adenocarcinoma (PDAC), macrophage-secreted basic fibroblast growth factor (bFGF) upregulates the expression of GFAP in SCs via the PI3K/Akt/c-myc pathway, thereby promoting the reprogramming of these cells [40].

The potential contributions of other immune and stromal cell types to SC activation and reprogramming in the TME have yet to be fully elucidated and represent promising areas for further investigation. These findings underscore the dynamic interplay between SCs and the TME, highlighting the importance of understanding these interactions for the development of novel therapeutic strategies that could harness or modulate SC functions to block cancer progression.

## 3. The Role of SCs in Tumor Immune Microenvironment

**Figure 2:**
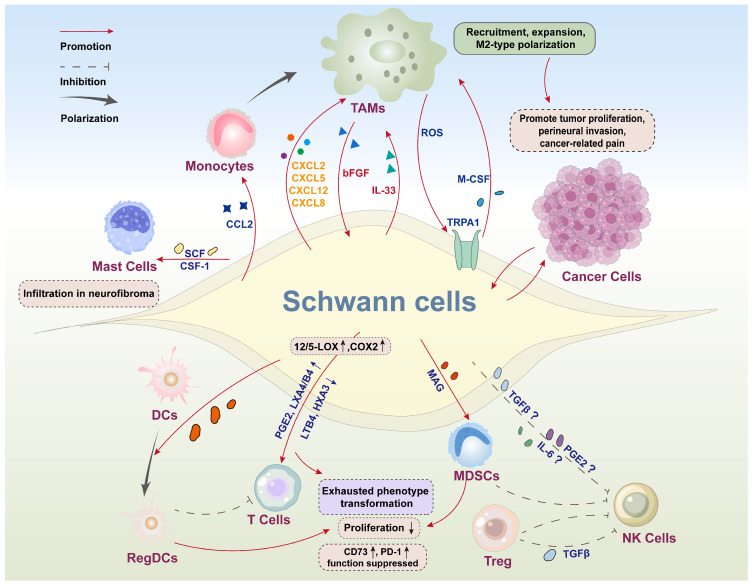
Schematic diagram illustrating the role of SCs in tumor immune microenvironment. SCs orchestrate an immunosuppressive TME and promote tumor proliferation, perineural invasion (PNI), and cancer-related pain by modulating immune cells. Through the secretion of various chemokines, cytokines, and other effector molecules, such as C-X-C chemokine ligand 12 (CXCL12), C-C chemokine ligand 2 (CCL2), macrophage colony-stimulating factor (M-CSF), and interleukin-33 (IL-33), SCs influence immune cells-mediated anti-tumor immunity through the following pathways: (1) promoting the polarization of immune cells such as tumor-associated macrophages (TAMs), dendritic cells (DCs) and T lymphocytes into certain protumorigenic cell subsets; (2) facilitating the activities of immune inhibitory cells in terms of recruitment, activation and immunosuppressive effects including M2-type TAMs, regulatory DCs (regDCs), and myeloid-derived suppressor cells (MDSCs); and (3) potentially restricting the cytotoxicity of natural killer (NK) cells. Additionally, TAMs can, in turn, exert a promoting effect on SC activation and function. Other abbreviations: PGE2: prostaglandin E2; LXA4/B4: lipoxins A4/B4; LTB4: Leukotriene B4; HXA3: Hepoxilin A3; MAG: myelin-associated glycoprotein; TGF-β: transforming growth factor-β; LOX: lipoxygenas; COX2: cyclooxygenase-2; SCF: stem cell factor; CSF1: colony-stimulating factor 1; bFGF: basic fibroblast growth factor; ROS: reactive oxygen species; TRPA1: transient receptor potential ankyrin 1; PD-1: programmed death-1; GFAP: glial fibrillary acidic protein. “↑” denotes upregulation; “↓” denotes downregulation; “?” denotes potential regulation.

SCs, the primary glial cells of the PNS, have emerged as significant players in the tumor–neuro–immune axis, regulating tumor development and progression [36,41]. It has been established that reprogrammed SCs secrete a diverse array of chemokines and cytokines to recruit and regulate immune cells, thereby creating an immunosuppressive microenvironment that is conducive to tumor progression [27,38,41]. These immune cells include tumor-associated macrophages (TAMs), natural killer (NK) cells, dendritic cells (DCs), T lymphocytes, myeloid-derived suppressor cells (MDSCs), and other immune cells (Figure 2). Different immune cells have varying impacts on tumor progression and the efficacy of tumor immunotherapy. Therefore, it is imperative to understand the interaction between SCs and immune cells, as well as the underlying mechanisms involved.

### 3.1. Regulatory Effects of SCs on TAMs

Macrophages within the TME are crucial immune cells that play a pivotal role in tumor progression, with their functions varying based on tumor type and stage. Initially, monocytes are recruited to the TME and differentiate into TAMs under the chemotactic effect of multiple cytokines secreted by various cells, including tumor cells, SCs, and several subsets of T cells [42,43,44,45,46,47]. Macrophages exhibit significant heterogeneity and are typically categorized into two major phenotypes: M1 and M2. M1 macrophages, activated by lipopolysaccharides and Th1 cytokines, possess anti-tumor properties. Conversely, M2 macrophages, stimulated by Th2 cytokines, exhibit immunosuppressive functions that support tumor growth and metastasis by promoting cell proliferation, immune suppression, and angiogenesis [48,49,50,51]. Most TAMs display an M2-like phenotype [52]. Notably, the phenotypic states of macrophage subpopulations are not static; microenvironmental signals dynamically regulate them, continuously adapting their functional phenotypes in response to these cues [49,53].

Numerous studies have demonstrated that interactions among SCs, macrophages, and cancer cells synergistically promote tumor cell proliferation, PNI, and cancer pain development [40,54,55,56]. The primary mechanism underlying this process is the ability of SCs to induce macrophage polarization toward the M2 phenotype, which is known for its pro-tumorigenic properties [40,54]. The mechanism by which SCs promote macrophage polarization involves the strategic secretion of various cytokines and chemokines, such as CCL2, CXCL5, CXCL12, and CXCL8 [54]. This, in turn, promotes cell proliferation in specific tumor types, such as lung cancer [54]. Other research has reported that SCs drive the recruitment of inflammatory monocytes from the circulation to the perineurium and promote their differentiation into macrophages by secreting CCL2 and activating the CCL2-CCR2 signaling pathway [43]. These macrophages subsequently produce cathepsin B, which disrupts the integrity of the nerve bundle membrane, facilitating tumor invasion into and along the nerve. Blocking the CCL2-CCR2 signaling pathway or cathepsin B significantly weakens PNI in vivo [36,43]. Recent research advances have highlighted a positive feedback loop involving bFGF/IL-33 between SCs and macrophages, particularly TAMs, which play a crucial role in the PNI process. As previously described, TAMs activate SCs through the bFGF/PI3K/Akt/c-myc/GFAP pathway. The activated SCs, in turn, secrete IL-33, which recruits macrophages to the neural periphery and induces their polarization toward the M2 phenotype, forming a positive feedback loop that promotes the invasion of cancer cells along the nerves [40].

Cancer-related pain is a significant contributor to the poor quality of life in cancer patients. The interaction between macrophages and SCs plays a vital role in the feed-forward pathway perpetuating cancer pain. In melanoma and lung cancer pain models, cancer cells were injected into the plantar region of the mouse’s right hind paw, simulating behavioral and functional changes akin to those observed in metastatic bone cancer pain. This study demonstrated that cancer-induced oxidative stress activates transient receptor potential ankyrin 1 (TRPA1) on SCs, triggering the release of macrophage colony-stimulating factor (M-CSF). This, in turn, drives the expansion of resident macrophages (rMΦ) within the sciatic nerve trunk. The amplified rMΦ population sustains this pathological feedback loop through its own oxidative stress, maintaining allodynia and spontaneous pain [55]. In another study, perineural breast cancer cell implantation induced macrophage infiltration into the sciatic nerve, accompanied by mechanical hypersensitivity and persistent spontaneous nociception. Specifically, redox effector-1 (Ref-1), secreted by breast cancer cells, was found to activate the secretion of the chemokine CXCL2 in SCs via the hypoxia-inducible factor (HIF)-1α/ROS pathway [56]. This cascade facilitated macrophage infiltration into the sciatic nerve, subsequently triggering spontaneous nociception or pain perception in the affected area. Notably, the study revealed that the macrophages infiltrating the sciatic nerve were mainly derived from peripheral blood circulation rather than from the proliferation of rMΦ [56]. Depleting hematopoietic macrophages using liposome-encapsulated clodronate significantly reduced pain perception and F4/80-positive macrophages in sciatic nerves [56]. These studies indicate that communication between macrophages and SCs may be an essential process in cancer-induced pain-like behaviors, highlighting how oxidative stress, SC-derived factors, and macrophage infiltration increase the sensitivity of signals within the sciatic nerve and amplify pain signaling in cancer models. However, while these models effectively replicate cancer-related pain in several cancer types, the lack of orthotopic implantation limits their clinical relevance. Future studies should prioritize orthotopic models to better simulate tumor-SC-immunocyte interactions in vivo.

### 3.2. Regulatory Effects of SCs on NK Cells

- NK cells are cytotoxic lymphocytes integral to the innate immune system. They are renowned for their ability to autonomously recognize and rapidly eliminate a wide range of harmful cells, playing a critical role in defense against microbial infections and anti-tumor immunity [57,58]. However, in the immunosuppressive TME, the balance of activating and inhibitory receptors expressed by NK cells is disrupted due to direct inhibitory effects exerted by multiple cell types or the secretion of inhibitory factors, leading to impaired NK cell cytotoxicity [58].
- Although direct interactions between tumor-associated SCs and NK cells have not been extensively studied, there is considerable crosstalk between NK cells and various cell populations within the TME [59,60,61]. SCs express several molecules related to NK cell dysfunction, such as TGF-β, PGE2, and IL-6, suggesting a potential relationship between SCs and NK cells [38,62,63,64,65].
- Recent studies have demonstrated that tumor-associated SCs secrete elevated levels of IL-6 across various tumor types, which is linked to immune regulation, tumor progression, and the perception of injury [38,64,65]. It has been established that IL-6 can inhibit the cytotoxicity of NK cells by activating the STAT3 signaling pathway, which mediates the downregulation of critical activating receptors on the surface of NK cells, such as NKp30 and NKG2D [58,66]. Based on these findings, we hypothesize that SCs may modulate NK cell function by secreting IL-6.
- In pancreatic cancer, SCs enhance tumor aggressiveness in a TGF-β-dependent manner [63]. Similarly, in melanoma, TGF-β levels secreted by SCs exposed to a tumor-conditioned medium are significantly upregulated [38]. TGF-β has been shown to reduce the expression of activating molecules on NK cells, thereby inhibiting their anti-tumor activity in various tumor types [67,68,69,70]. Consequently, it is plausible that TGF-β secreted by SCs also regulates NK cell function.
- Moreover, melanoma-reprogrammed SCs increase the production of anti-inflammatory metabolites such as prostaglandin E2 (PGE2), which suppress anti-tumor T-cell responses [62]. Importantly, PGE2 has been implicated in promoting tumor immune escape by inhibiting NK cell cytotoxicity and modulating their phenotype, particularly in thyroid and lung cancers [71,72]. Therefore, it is reasonable to propose that PGE2 secreted by SCs may similarly affect NK cell function.
- In this discussion, we have highlighted several factors secreted by SCs that are relevant to NK cell function. These findings highlight the potential of SCs to modulate NK cell responses through the secretion of immunomodulatory cytokines. Future research should focus on elucidating the mechanisms underlying the crosstalk between SCs and NK cells within the TME, as these interactions may reveal novel therapeutic targets for enhancing NK cell function in cancer treatment.

### 3.3. Regulatory Effects of SCs on DCs

As initiators and coordinators of adaptive immune responses, DCs have great potential to induce effective anti-tumor immunity, leading to the development of various therapeutic strategies targeting DCs in cancer [73]. Within the TME, DCs present major histocompatibility complex (MHC) molecules on tumor-associated antigens and provide co-stimulatory molecules and soluble factors to attract and mediate the activation and function of anti-tumor T cells [73].

Several studies have shown that SCs orchestrate an immunosuppressive TME by polarizing and regulating the activity of DCs. In a mouse melanoma model, tumor-activated SCs profoundly influence DCs, steering them toward a regulatory phenotype. When SCs are co-cultured with melanoma cells, they notably amplify the chemotactic attraction of CD11c^+^ DCs. Remarkably, DCs treated with melanoma-activated SCs lose their capacity to induce the proliferation of activated T cells, effectively dampening T-cell proliferation in an in vitro setting [27].

Furthermore, the selective genetic elimination of endogenous SCs precipitates a significant increase in DC infiltration within the tumor [74]. These infiltrating DCs activate cytotoxic T cells, which play a pivotal role in the direct eradication of cancer cells [74]. While the underlying mechanism was not elucidated in this study, we speculate that SC ablation may improve the immunosuppressive TME associated with SCs. Additionally, it could serve as a “signal of injury”, triggering an acute inflammatory response, thus indirectly promoting the augmented presence of functional DCs. These findings underscore the pivotal role of SCs in modulating the TME, specifically in shaping the function of DCs, which in turn impacts the activation and expansion of T cells, which are integral to the adaptive immune response against cancer.

### 3.4. Regulatory Effects of SCs on T Lymphocytes

T cells are critical components of effective anti-tumor immunity and sustained immune responses, encompassing various subpopulations such as regulatory T cells, helper T cells, and cytotoxic T cells [75]. Despite the robust infiltration of T cells into tumor sites, these cells often become dysfunctional within the TME, adopting exhausted and senescent phenotypes that hinder effective anti-tumor immunity and immunotherapy while maintaining an immunosuppressive TME [76].

Recent studies have highlighted SCs’ ability to suppress T-cell function through various direct and indirect mechanisms. Similar to macrophages, T cells exhibit distinct functional phenotypes as well. GV. Shurin et al. [77] reported that SCs can induce the polarization of tumor-associated T cells. Specifically, melanoma-derived TGF-β acts as a potent activator of SCs via the SMAD and MAPK/ERK signaling pathways. Upon activation, SCs become prolific producers of prostaglandin E2 (PGE2), a lipid mediator that exerts a potent suppressive effect on T-cell proliferation by specifically inhibiting CD3/CD28-induced T-cell proliferation [68]. Furthermore, this signaling cascade initiated by melanoma-derived TGF-β prompts SCs to upregulate the expression of CD73 and programmed death-1(PD-1) on CD4^+^ and CD8^+^ T cells, thereby inhibiting T-cell function and driving their polarization toward an exhausted phenotype [68]. CD73 facilitates the conversion of extracellular adenosine, a molecule known for its immunosuppressive properties, while PD-1 serves as a checkpoint inhibitor that normally prevents autoimmunity but, in this scenario, contributes to T-cell exhaustion. This intricate web of interactions orchestrated by SCs under the influence of T-cell exhaustion underscores the complexity of the TME and its role in evading immune surveillance.

Further investigations revealed that SCs in the skin surrounding melanoma undergo reprogramming into reparative immunosuppressive cells with dysregulated lipid oxidation [62]. These peritumoral skin SCs upregulate 12/15-lipoxygenase and COX-2, leading to the altered proportion of polyunsaturated fatty acid metabolites. This metabolic reprogramming results in a marked increase in the production of anti-inflammatory mediators, such as PGE2 and lipoxin A4/B4, and a decrease in pro-inflammatory products, such as leukotriene B4 and hepoxilin A3. Consequently, these changes inhibit the activity of anti-tumor effector T cells, thereby modulating both systemic and local anti-tumor immune responses [62].

In summary, SCs facilitate a pro-cancer phenotypic shift in T cells and inhibit the activity of effector T lymphocytes, contributing to the immunosuppressive environment of the TME.

### 3.5. Regulatory Effects of SCs on MDSCs

MDSCs are a heterogeneous population of immature myeloid cells that become pathologically activated due to persistent inflammatory signals, causing them to deviate from normal differentiation [78]. Characterized by anti-inflammatory and immunosuppressive functions, MDSCs play a crucial role in tumor development, metastasis, and treatment resistance by directly supporting tumors’ immune escape [78,79].

Recent research has revealed that melanoma-activated SCs chemoattracted MDSCs and significantly increased their ability to suppress T-cell proliferation in vitro, indicating that melanoma-activated SCs augment the immunosuppressive activity of these MDSCs. Further studies identified that myelin-associated glycoprotein (MAG) overexpressed by tumor-treated-SCs was responsible for this phenomenon. MAG is a type I transmembrane glycoprotein known to negatively regulate axonal growth. Both melanoma-activated SCs with high MAG expression and recombinant MAG independently increased the immunosuppressive activity of MDSCs in T-cell inhibitory assay [41].

Collectively, these findings suggest that SCs actively contribute to the recruitment and functional modulation of MDSCs within the tumor milieu, thereby shaping an immunosuppressive and tolerogenic immune landscape that facilitates tumor immune escape.

### 3.6. Regulatory Effects of SCs on Other Immune Cells

Other immune cells, such as mast cells, also interact with SCs. This interaction is particularly notable in neurofibromas, benign tumors primarily composed of SCs and fibroblasts, where mast cell infiltration is a common pathological feature. Liao et al. proposed a hypothetical model for the initiation and tumorigenesis of neurofibromas, suggesting that NF1-mutated SCs interact with the inflammatory microenvironment to promote neurofibromagenesis. In this model, tumorigenic SCs express chemokines and cytokines, such as stem cell factor (SCF) and colony-stimulating factor 1 (CSF1), which recruit inflammatory cells, including mast cells and macrophages, thereby sustaining tumorigenesis [80]. Notably, the association between SCs and mast cells in peripheral cancers remains unexplored.

## 4. Therapeutic Strategies for Targeting SCs to Enhance Anti-Cancer Immune Responses

With the advancing understanding of the tumor-SCs-immune axis, SCs are emerging as promising therapeutic targets for cancer treatment. In recent decades, preclinical studies aiming to restore anti-cancer immune responses through SC-targeted therapies have increased significantly. The primary strategies for SC-based immunotherapy may include inhibiting SC reprogramming, disrupting SC communication with tumor-associated immune cells, and the targeted elimination of SCs within the TME.

### 4.1. Inhibition of SCs Reprogramming

As discussed previously, reprogrammed SCs within the TME exhibit enhanced immunosuppressive effects and contribute more significantly to tumor progression than control SCs. Therefore, inhibiting SC reprogramming represents a promising strategy for targeted therapy. For instance, TAMs activate SCs through the bFGF/PI3K/Akt/c-myc/GFAP signaling pathway. To counteract the activation of this pathway, the development of inhibitors targeting PI3K, Akt, and FGFR antagonists could be a potential therapeutic approach [40]. Such strategies are expected to prevent PNI, thereby reducing neuralgia and slowing tumor progression in patients with pancreatic ductal adenocarcinoma (PDAC) [40].

On the other hand, Deborde et al. [31] considered c-Jun as a key regulator of this cancer-associated SC and illustrated that reprogrammed SCs could promote pancreatic cancer cell migration and invasion. Moreover, SP600125, a c-Jun N-terminal kinase (JNK) inhibitor [31], was found to influence c-Jun activity in SCs and to impede PNI in vivo, demonstrating the potential for targeted therapies in pancreatic cancer.

Melanoma also induces the reprogramming of SCs to promote tumor growth [38]. Similar results have been observed in cervical cancer [81], colorectal cancer [82] and cholangiocarcinoma [34]. Given the significance of the tumor-nerve system axis in various cancers, there is an urgent need to develop SC-specific inhibitors to interrupt the reprogramming of SCs.

### 4.2. Disrupting SC Communication with Tumor-Associated Immune Cells

Considering that SCs secrete various cytokines and chemokines to regulate the phenotypes and functions of immune cells, specific drugs or biologics could be developed to disrupt their secretory functions [40,56,83]. Such interventions could inhibit the paracrine effects of SCs on cancer-promoting immune cells, prevent the conversion of immune cells to a cancer-promoting phenotype, and restore the normal anti-tumor functions of immune cells [54,62,77]. Additionally, therapeutic strategies based on RNA interference technology or gene-editing technology show promise for directly targeting key signaling pathways or gene expression in SCs to disrupt their interactions with tumor-associated immune cells. For example, one study demonstrated that PDAC cells secrete NGF to induce autophagy in SCs, which in turn promotes PNI. Knockdown of NGF was shown to partially reverse this phenomenon, suggesting that targeting NGF or SC autophagy could block PNI, providing a novel treatment approach [84].

While these strategies are promising, they remain speculative. Future research should focus on developing therapies aimed at reducing or blocking SC secretion that influences immune cells and promotes tumor progression.

### 4.3. Targeting Elimination of SCs in the TME

In a transgenic mouse model of pancreatic cancer, specific depletion of SCs through intrapancreatic in situ injection of the adeno-associated virus (AAV)-expressing, the GFAP-driven diphtheria toxin A (DTA) gene significantly inhibited tumorigenesis. Concurrently, depletion of SCs in vivo using small molecule compounds fluorocitrate significantly inhibited tumor growth, enhanced the infiltration and cytotoxicity of CD8^+^ T cells, and improved the sensitivity of PDAC to immunotherapy [85]. Additionally, enteric glial cells, which are glial cells of the PNS like SCs and originate from neural crest cells, show similar results [86]. Depleting GFAP^+^ enteric neuroglia in an azoxymethane/dextran sodium sulfate (AOM/DSS)-induced mouse model of colorectal cancer significantly reduced tumor load and precancerous lesions. Similarly, depletion of enteric neuroglia in a mouse model of familial adenomatous polyposis led to a notable reduction in the number of adenomas [86].

R. L. Bakst et al. demonstrated that radiotherapy-induced SC death is a critical factor in PNI impairment. Lower doses of radiotherapy could be applied to the neural portion of the peri-neural area surrounding the tumor to induce SC death [43]. However, controlling the damage caused by radiotherapy to normal nerve function requires further study. Nonetheless, the concept of selectively targeting and eliminating SCs in the TME emerges as a promising avenue for therapeutic intervention. This strategy capitalizes on the unique vulnerabilities of SCs in the context of cancer, aiming to disrupt the supportive role these cells play in tumor progression and potentially enhance the efficacy of existing cancer treatments.

## 5. Conclusions

Due to the crucial role of SCs in tumor initiation and progression within the TME, SCs have garnered increasing attention over the past decades. This paper elaborates on the interactions between SCs and infiltrating immune cells within the TME and the underlying mechanism. SCs have been shown to influence the phenotype and function of immune cells directly, thus promoting the formation of an immunosuppressive TME. This effect is primarily mediated through their secretory functions and operates via three main mechanisms: (i) driving immune cells such as TAMs, mast cells, DCs, and T lymphocytes to polarize into pro-tumor subtypes or functionally exhausted phenotypes; (ii) promoting the recruitment, infiltration, activation, and immunosuppressive behavior of immune-suppressor cells, including TAMs, DCs and MDSCs; and (iii) potentially reducing the cytotoxic activity and cytokine secretion of immune effector cells like NK cells. Notably, certain infiltrating immune cells, such as TAMs, can reciprocally enhance the activation and function of SCs. These interactions form an immunosuppressive loop, further reinforcing the immunosuppressive nature of the TME.

Considering the intricate immunosuppressive functions that SCs execute within the TME, the precise elimination of SCs presents a promising frontier for enhancing anti-tumor therapies. These targeted interventions aim to recalibrate the immunological balance tipped by SCs, thereby potentiating anti-tumor immunity. However, since SCs are widely present in the human PNS, it is a great challenge to target SCs to treat tumors while protecting normal neurological functions, which requires more exploratory research.

## Abbreviations

SCs: Schwann cells; PNI: perineural invasion; TME: tumor microenvironment; NK: natural killer; DCs: dendritic cells; MDSCs: myeloid-derived suppressor cells; PDAC: pancreatic ductal adenocarcinoma; GFAP: glial fibrillary acidic protein; TAMs: tumor-associated macrophages; CCL: C-C motif chemokine ligand; CCR: C-C motif chemokine receptor; CXCL: C–X–C motif chemokine ligand; EMT: epithelial–mesenchymal transition; TGF-β: transforming growth factor-β; IL: interleukin; PGE2: prostaglandin E2; PNS: peripheral nervous system; ECM: extracellular matrix; rMΦ: resident macrophages; TRPA1: transient receptor potential ankyrin 1; OSCC: oral squamous-cell carcinoma; MAG: myelin-associated glycoprotein; NGF: nerve growth factor; ROS: reactive oxygen species; Ref-1: redox effector-1;HIF-1α: hypoxia-inducible factor-1α; M-CSF: macrophage colony-stimulating factor; NO:nitric oxide; MHC: major histocompatibility complex; PD-1: programmed death-1; SCF: stem cell factor; CSF1: colony-stimulating factor 1; COX-2: cyclooxygenase-2; AOM/DSS: azoxymethane/dextran sodium sulfate; AAV: adeno-associated virus; DTA: diphtheria toxin A; bFGF: basic fibroblast growth factor; LTB4: leukotriene B4; HXA3: hepoxilin A3; LOX: lipoxygenase.
